# Learning-induced uncertainty reduction in perceptual decisions is task-dependent

**DOI:** 10.3389/fnhum.2014.00282

**Published:** 2014-05-07

**Authors:** Feitong Yang, Qiong Wu, Sheng Li

**Affiliations:** ^1^Department of Psychology, Peking UniversityBeijing, China; ^2^Department of Psychological and Brain Sciences, Johns Hopkins UniversityBaltimore, MD, USA; ^3^Department of Human Sciences, College of Education and Human Ecology, Ohio State UniversityColumbus, OH, USA; ^4^Key Laboratory of Machine Perception (Ministry of Education), Peking UniversityBeijing, China; ^5^PKU-IDG/McGovern Institute for Brain Research, Peking UniversityBeijing, China

**Keywords:** uncertainty, learning, perceptual decision, categorization, Glass pattern

## Abstract

Perceptual decision-making in which decisions are reached primarily from extracting and evaluating sensory information requires close interactions between the sensory system and decision-related networks in the brain. Uncertainty pervades every aspect of this process and can be considered related to either the stimulus signal or decision criterion. Here, we investigated the learning-induced reduction of both the signal and criterion uncertainty in two perceptual decision tasks based on two Glass pattern stimulus sets. This was achieved by manipulating spiral angle and signal level of radial and concentric Glass patterns. The behavioral results showed that the participants trained with a task based on criterion comparison improved their categorization accuracy for both tasks, whereas the participants who were trained on a task based on signal detection improved their categorization accuracy only on their trained task. We fitted the behavioral data with a computational model that can dissociate the contribution of the signal and criterion uncertainties. The modeling results indicated that the participants who were trained on the criterion comparison task reduced both the criterion and signal uncertainty. By contrast, the participants who were trained on the signal detection task only reduced their signal uncertainty after training. Our results suggest that the signal uncertainty can be resolved by training participants to extract signals from noisy environments and to discriminate between clear signals, which are evidenced by reduced perception variance after both training procedures. Conversely, the criterion uncertainty can only be resolved by the training of fine discrimination. These findings demonstrate that uncertainty in perceptual decision-making can be reduced with training but that the reduction of different types of uncertainty is task-dependent.

## Introduction

Perceptual decision-making plays important roles in our daily life. However, uncertainty pervades effective completion of this process in many aspects. In previous investigations of perceptual decision-making, uncertainty was frequently regarded as one general factor of the decision process (Huettel et al., 2005; Grinband et al., 2006; Heekeren et al., 2008; Kepecs et al., 2008; Barthelme and Mamassian, 2009; Kiani and Shadlen, 2009; Daniel et al., 2011; de Gardelle and Summerfield, 2011), but see Michael et al. (2013). The lack of discrimination between different types of uncertainty may impede our understanding of the underlying mechanisms of perceptual decisions. In traditional literature of perceptual categorization, a decision boundary or a number of prototypes were assumed, and the comparison with the boundary or prototypes was the key process for correctly categorizing new examples (Medin and Schwanenflugel, 1981; Ashby and Gott, 1988; Maddox and Ashby, 1993; Love et al., 2004). In this way, uncertainty of making correct categorization is correlated with the distance between a specific stimulus and the categorical boundary (Kepecs et al., 2008). On the other hand, uncertainty level can also be manipulated by varying the signal-to-noise ratio of a stimulus (Kiani and Shadlen, 2009). Here, we aimed to understand different types of uncertainty by manipulating different stimulus parameters in two visual categorization tasks. We examined the signal uncertainty, which stemmed from different signal strengths in a noisy background; the criterion uncertainty, which was due to insufficient amount of knowledge about the categorization criterion; and their interactions. This dichotomy on uncertainty mirrors the classification of internal vs. external uncertainty in literature of social and economic judgments suggesting that decision uncertainty could originate from the environmental dispositions that we cannot control or from the ignorance or insufficient knowledge that could be controlled by the decision makers (Howell, 1971; Kahneman and Tversky, 1982; Volz et al., 2004; Hsu et al., 2005).

Perceptual decision is believed to be a multi-stage process, including but not limited to sensory evidence collection and accumulation, criterion comparison, performance monitoring, and action execution (Shadlen and Newsome, 2001; Mazurek et al., 2003; Heekeren et al., 2008). The information processing of different perceptual decision tasks can vary at different stages. Therefore, it is reasonable to expect that the neural representation of decision uncertainty is also task-dependent and can be attributed to different stages of decision-making. In fact, our previous functional magnetic resonance imaging (fMRI) study investigated uncertainty modulation in two perceptual decision tasks, and we demonstrated the task-dependent uncertainty modulation in the human brain (Li and Yang, 2012). In this study, the participants performed two categorization tasks that required either fine discrimination (i.e., the criterion comparison task) or signal extraction (i.e., the signal detection task). The criterion comparison task required participants to compare clear global patterns with an implicit decision boundary defined by experimenter (Li et al., 2009, 2012). In the signal detection task, the participants were required to extract the global form from its noisy background (Mayhew et al., 2012). We identified the areas responsible for performance monitoring, such as the posterior medial frontal cortex (pMFC), as the common hubs for representing uncertainty modulation (Ridderinkhof et al., 2004). Importantly, we also identified dissociable cortical networks that were correlated with uncertainty modulation in different tasks. In the criterion comparison task, uncertainty modulated the fMRI activity of areas related to rule retrieval, whereas in the signal detection task, uncertainty modulated the fMRI activity of higher visual areas.

Previous studies have shown that perceptual training is known to improve the performance of perceptual decisions (Sagi and Tanne, 1994; Ghose, 2004; Sasaki et al., 2010). Investigating the effect of perceptual training can also inform the mechanisms underlying the decision-making process. The relationship between perceptual training and uncertainty reduction of perceptual decisions is an interesting issue to address. Particularly, understanding the task-dependency of the reduction of different types of uncertainty is critical for the evaluation of perceptual training efficiency. Dosher and Lu (2005) have shown that the ability to filter external noise in stimuli can be improved by training on both the clear and noisy displays in a Gabor orientation discrimination task. However, only training effect on the clear displays can be generalized to the noisy displays, but not vice versa. The asymmetric transfer of training effect was attributed to the limited enhancement of stimulus signal in neural system when training was applied to the noisy displays, as amplifying the stimulus would amplify the signal and external noise together (Dosher and Lu, 1998, 2005). Nevertheless, whether their results can be generalized to high level visual perception, such as pattern categorization, and how the uncertainty on decision criterion changes with training remain less well-understood. To investigate the training effect on uncertainty reduction in the present study, we trained the participants on either the criterion comparison task or the signal detection task and tested their behavioral performance on both tasks after the training. Moreover, we fitted the behavioral data with a model that incorporated both the criterion and signal uncertainties. Our results showed that the learning effect indexed as the categorization accuracy transferred from the criterion comparison task to the signal detection task, but not vice versa. Furthermore, the results from the model fitting revealed that the signal uncertainty could be reduced by training in both tasks, but the reduction of criterion uncertainty was observed only after training in the criterion comparison task.

## Methods

### Participants

Twenty six (10 males, mean age: 21.6, range: 18–25 years) right-handed, healthy students from Peking University participated in the study. All participants had normal or corrected to normal vision and gave written informed consent. The experiment was approved by the local ethics committee. All participants were paid equally for their participation.

### Stimuli

Glass patterns were used as stimuli in the experiment (Li and Yang, 2012). Each pattern consisted of 600 white dipoles randomly distributed in a square aperture (7.3° × 7.3°) on a black background. The distance between the two dots in a dipole was 15.4 arc min, and each dot was one pixel in size. For each dot dipole, the spiral angle was defined as the angle between the hidden line linking the two dots of the dipole and the radius from the center of the stimulus aperture to the center of the dipole. The proportion of dipoles aligned according to a specified spiral angle (i.e., the signal dipoles) was defined as the signal level for each stimulus. The spiral angles were randomly assigned for the noise dipoles. The global percept of a Glass pattern was determined by the spiral angle of the signal dipoles. As the spiral angle increased from 0° to 90°, the global percept of the pattern gradually changed from radial to concentric.

**Figure 1:**
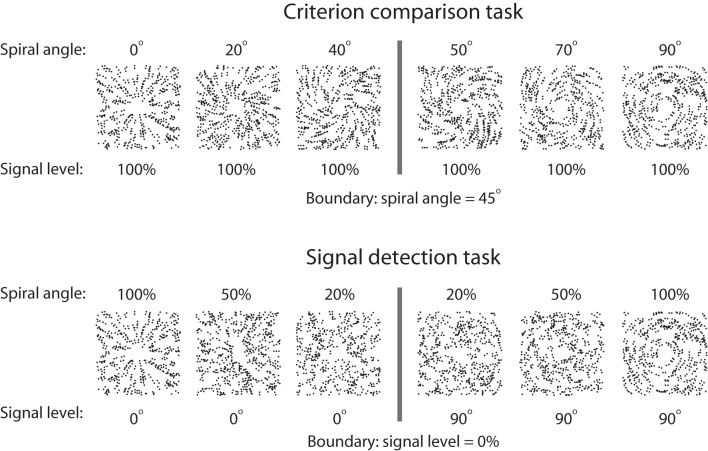
**Stimulus sets for the criterion comparison and signal detection tasks**. For the criterion comparison task, the signal level of the stimuli was set to 100%, and the spiral angles varied from 0° to 90°. For the signal detection task, Glass patterns of 0° (radial) and 90° (concentric) spiral angles were presented, and the signal level varied between 0 and 100%. Modified from Li and Yang (2012).

By manipulating the spiral angle and the signal level, we constructed two stimulus sets (Figure 1). For the criterion comparison set, stimuli were generated between radial and concentric patterns by parametrically varying the spiral angles from 0° (radial pattern) to 90° (concentric pattern). All stimuli were presented at the 100% signal level. For the signal detection set, perceptual uncertainty was created by manipulating the signal-to-noise ratio. Thus, stimuli were presented at either 0° (radial pattern) or 90° (concentric pattern) spiral angles, and the signal level ranged from 0 to 100%. The criterion uncertainty was operationally defined as the angular difference between the presented stimulus and the decision boundary (i.e., the criterion to be compared). The signal uncertainty was operationally defined as the noise level for the given stimulus. Thus, the sources of decision uncertainty for the criterion comparison and signal detection tasks mainly originated from the criterion and signal uncertainties. We specifically selected parameter levels for each task. In the criterion comparison task, we selected ten levels of spiral angles: 23, 32, 38, 41, 43, 47, 49, 52, 58, and 67°. In the signal detection task, we selected 10 different levels: radial patterns at 5%, 9%, 16%, 28%, 48% signal strength, and concentric patterns at 5%, 9%, 16%, 28%, 48% signal strength. These parameter levels are chosen based on pilot experiment results so that uncertainty levels matched in difficulty between different tasks.

### Procedure

Participants were randomly assigned to either the criterion comparison group, in which they were trained on the categorization task based on the criterion comparison stimulus set, or to the signal detection group, in which they were trained on the categorization task based on the signal detection stimulus set. Each participant successively participated in a 1-day pre-test session, a 3-day training session, and a 1-day post-test session. During the training session, the participant was trained on the categorization task only based on the stimulus set he/she was assigned. During the test sessions, the participant performed the categorization task on both stimulus sets. From the perspective of the participants, they performed the same task based on two stimulus sets. They were instructed to perform a categorization task to assign the Glass patterns into either radial or concentric categories.

### Pre-test and post-test sessions

Before the pre-test session, each participant was presented with two 100% signal level Glass patterns (0° and 90° spiral angle). The participant was instructed to categorize the stimuli as either radial or concentric, and they performed 40 practice trials on each stimulus set. Auditory feedback was given only when the participant incorrectly categorized a stimulus. The center of the stimulus space (i.e., 45° spiral angle) was defined as the categorical boundary.

Each test session consisted of four blocks with two blocks for each stimulus set. The order of the blocks was randomized. Each block consisted of 120 trials. For the criterion comparison task, the participant was presented with 100% signal level Glass patterns at different spiral angles. For the signal detection task, the participant was presented with 0° and 90° Glass patterns at different signal levels. These stimulus conditions were selected to generate a base-10 logarithmic stimulus space in which the uncertainty increased with equal step size from the center of the space. Each trial started with a 300-ms fixation period followed by a 200-ms stimulus presentation period. The trial ended once the participant responded to the stimulus. No feedback was provided. There was a 400-ms inter-trial interval.

### Training sessions

We adopted a QUEST procedure (Watson and Pelli, 1983) for the training sessions. Each training session consisted of 20 blocks of the assigned task. Each block started with a 1500-ms fixation period, which was followed by 80 trials. Each trial started with a 200-ms stimulus presentation period and was followed by a maximum 1500-ms blank screen for their response period. The observers received auditory feedback following incorrect choices. There was a 300-ms interval between each trial. We intermixed two sets of QUEST processes within each block. Each QUEST process consisted of 40 trials, and its parameters of interest (spiral angle for the criterion comparison task and signal level for the signal detection task) for the radial patterns and the concentric patterns were adjusted separately. Specifically, for the criterion comparison task, one QUEST process adjusted the stimulus' spiral angle from 0° to 45° for radial patterns, and the other QUEST process adjusted the spiral angle from 90° to 45° for concentric patterns. For the signal detection task, one QUEST process adjusted the stimulus' signal level from 100 to 0% for the radial patterns, and the other QUEST process adjusted the signal level from 100 to 0% for the concentric patterns. In each trial, one of the two QUEST processes, which were for the radial and concentric patterns, was randomly selected. A corresponding stimulus was presented according to the chosen QUEST process. This QUEST process was then updated based on the participant's categorization response. For the criterion comparison task, the stimulus following the response would be closer to 45° if the response was correct or be away from 45° if the response was wrong. For the signal detection task, the stimulus following the response would be lower in signal strength if the response was correct or be higher in signal strength if the response was wrong. Therefore, in each block, the two QUEST processes updated independently. This procedure ensured that the parameters of the stimuli were adjusted according to the participant's performance, and the training load was maintained at the same level for all participants across all training sessions and tasks.

### Computational model

**Figure 2:**
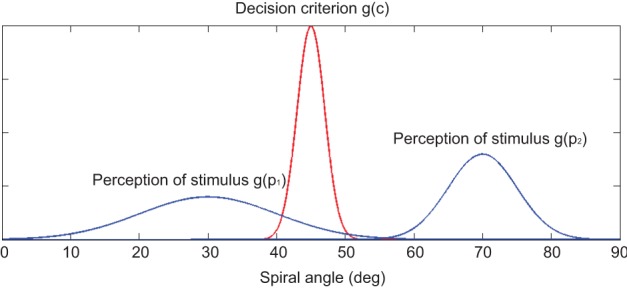
**Schematic illustration of the computational model**. An example of the Double Model is shown here. The decision process can be considered as comparing the perceived spiral angle of a stimulus (*p*_*i*_) with the implicit decision criterion (*c*_*i*_). If *p*_*i*_ > *c*_*i*_, the stimulus was categorized as a concentric pattern. Otherwise, if *p*_*i*_ < *c*_*i*_, the stimulus was categorized as a radial pattern. The red curve represents the decision criterion. The two blue curves represent the perceived spiral angle of two stimuli with different signal strengths: the left blue curve represents the perception of a low-signal-strength Glass Pattern whose spiral angle was 30°, and the right blue curve represents the perception of a high-signal-strength Glass Pattern whose spiral angle was 70°.

We modeled the perceptual decision process in our experiment with a model that incorporated both the criterion and signal uncertainties (Kepecs et al., 2008). In the model, we assumed that there was an implicit decision criterion of spiral angle (*c*_*i*_) that represented the boundary threshold for a participant during the categorical decision (Figure 2). The decision process can be considered as comparing the perceived spiral angle of a stimulus (*p*_*i*_) with the implicit decision criterion. If *p*_*i*_ > *c*_*i*_, the stimulus was categorized as a concentric pattern. Otherwise, if *p*_*i*_ < *c*_*i*_, the stimulus was categorized as a radial pattern. To account for the trial-by-trial variability of perception, for each trial *p*_*i*_ was drawn from a Gaussian distribution, g(*p*_*i*_), centered at the spiral angle of the presenting stimulus. The variance of g(*p*_*i*_) represented the signal uncertainty for the specific signal level of the stimulus. Additionally, to account for the trial-by-trial variability of decision criterion, *c*_*i*_ was drawn from a Gaussian distribution, g(*c*_*i*_). This variance represented the criterion uncertainty, and the mean determined the decision bias: the larger the mean, the higher the probability of reporting radial category. Alternatively, we also considered a situation in which *c*_*i*_ was a single value that was not sampled from a Gaussian distribution (i.e., no criterion uncertainty).

We fitted the models with behavioral data in the pre-test and post-test sessions separately. For each session, we fitted the model with the combined behavioral data from both the criterion comparison and signal detection tasks. We assumed that the variance of g(*p*_*i*_) varied across signal levels and test sessions due to training. The variance of g(*p*_*i*_) for the stimuli at a given signal level remained the same within each test session. However, the mean and variance of g(*c*_*i*_) varied only between the pre-test and post-test sessions but remained the same within each test session. We fitted the variances of g(*p*_*i*_) in two ways: (1) Full Model: the variance of each signal level was fitted independently; or (2) Simple Model: the variance for all signal levels was fitted together with an exponential decay function of signal level (*s*) (please refer to the footnote for the reason of choosing exponential function^1^): σ = α*e*^−β × *s*^, where σ was the variance of the distribution, and α and β were fitted based on the behavioral data. Together with the alternative choices of fitting the criterion uncertainty (variance of g(*c*_*i*_), Double Model) or not (Single Model), we compared four candidate models: Double-Full Model, Double-Simple Model, Single-Full Model, and Single-Simple Model. Details of each model are given below.

#### Double-full model

Both the criterion uncertainty [variance of g(*c*_*i*_)] and signal uncertainty [variance of g(*p*_*i*_)] were fitted in the model. The signal uncertainty was fitted for each signal level independently. There were eight free parameters. Six of them were the variance of g(*p*_*i*_) that corresponded to the six signal levels of the stimuli. The other two free parameters were the mean and variance of g(*c*_*i*_) for the criterion uncertainty.

#### Double-simple model

In this model, both the criterion uncertainty and signal uncertainty were fitted. However, the signal uncertainty was fitted with an exponential decay function of signal strength. There were four free parameters in total: the α and β for the exponential decay function and the mean and variance of g(*c*_*i*_) for the criterion uncertainty.

#### Single-full model

Only the signal uncertainty was fitted in the model. The decision criterion *c*_*i*_ was a single value. There were seven free parameters: six of them for variance of g(*p*_*i*_) that corresponded to the six signal levels of the stimuli and one for the value of the decision criterion *c*_*i*_.

#### Single-simple model

Only the signal uncertainty with an exponential decay function of signal strength was fitted in the model. The decision criterion *c*_*i*_ was a single value. There were three free parameters: the α and β for the exponential decay function and the value of the decision criterion *c*_*i*_.

We fitted the candidate models with the Maximum Likelihood Estimation method. In each trial, a stimulus with a spiral angle θ_*i*_ and signal level *s*_*i*_ was presented. The perceived spiral angle p_*i*_ was a sample drawn from a Gaussian distribution whose mean was θ_*i*_ and whose variance was σ_*i*_, namely *p*_*i*_ ~ *N*(θ_*i*_, σ^2^_*i*_).

In the Single-Full Model and the Single-Simple Model, the decision criterion was a single value *c*_*i*_. If *p*_*i*_ > *c*_*i*_, the stimulus was categorized into a concentric group. Namely, the probability of reporting a concentric group was:

$$p(concentric)=\int_{c}^{90}\frac{1}{\sigma_{i}\sqrt{2\pi }}e^{-\frac{{\left(p_{i}-\theta_{i}\right)}^{2}}{2\sigma_{i}^{2}}dp_{i}}$$

In the Double-Full Model and Double-Simple Model, the perceived spiral angle *p*_*i*_ was a sample drawn from a Gaussian distribution as mentioned above. The decision criterion, *c*_*i*_, was also a sample drawn from a Gaussian distribution whose mean and variance were μ_*i*_ and δ_*i*_ respectively, namely *c*_*i*_ ~ *N*(μ_*i*_, δ^2^_*i*_). If *p*_*i*_ > *c*_*i*_, the stimulus was categorized into a concentric group. The probability of reporting a concentric group was:

$$p(concentric)=\int_{0}^{90}dc_{i}\int_{c_{i}}^{90}f(c_{i})g(p_{i})dp_{i}$$

where $f(c_{i})=\frac{1}{\delta_{i}\sqrt{2\pi }}e^{-\frac{{\left(c_{i}-\mu_{i}\right)}^{2}}{2\delta_{i}^{2}}}$, $g(p_{i})=\frac{1}{\sigma_{i}\sqrt{2\pi }}e^{-\frac{{\left(p_{i},-\theta_{i}\right)}^{2}}{2\sigma_{i}^{2}}}$

We simplified the above equation for the data fitting by converting the bivariate integration into a univariate integration according to the definition:

$$p\;(concentric)\;=p\;(p_{i}\;>\;c_{i})\;=p\;(c_{i}-p_{i}<0)$$

let *x* = *c*_*i*_ − *p*_*i*_ because *c*_*i*_ ~ *N*(μ_*c*_, δ^2^_*i*_) and *p*_*i*_ ~ *N*(θ_*i*_, σ^2^_*i*_), then we had *x* ~ *N*(μ_*i*_ − θ_*i*_, δ^2^_*i*_ + σ^2^_*i*_). Hence,

$$p\left(concentric\right)=p(x<0)=\int_{-90}^{0}h(x)dx$$

where $h\left(x\right)=\frac{1}{\sqrt{2\pi }\sqrt{\delta_{i}^{2}+\sigma_{i}^{2}}}e^{-\frac{{\left[x\;-\left(\mu_{i}-\theta_{i}\right)\right]}^{2}}{2(\delta_{i}^{2}+\;\sigma_{i}^{2})}}$.

We calculated Akaike's Information Criterion (AIC) and Bayesian Information Criterion (BIC) to compare the different models. These two criteria address the issue of over-fitting, the trade-off between each model's goodness of fit, and its complexity: *AIC* = −2 ln *MLE* + 2*k* and *BIC* = −2 ln *MLE* + *k* ln *N*, where *MLE* was the value of the maximum likelihood, *k* was the number of free parameters, and *N* was the number of trials used to fit the model. The model became better as the criteria value became smaller.

In summary, we fitted four alternative models with participants' behavioral data in the pre-test and post-test sessions. We performed the modeling analysis aiming to identify the model that can best characterize the behavioral performance among the four candidate models and to justify the existence of the two types of uncertainty. The best model was selected by comparing the AIC and BIC values of the candidate models. We also aimed to use the best model to explain the training effects on different categorization tasks. This was achieved by comparing the training induced changes in fitted model parameters [*g*(*p*_*i*_) and *g*(*c*_*i*_)] between the pre-test and post-test sessions across tasks and participant groups.

## Results

### Behavioral results

The accuracies of training sessions were approximately 80% correct across days and participant groups. A mixed-design analysis of variance [ANOVA, task (between participants) × training day (within participant)] on accuracy showed no significant main effects of task [*F*_(1, 24)_ = 3.40, *p* = 0.08], training day [*F*_(2, 48)_ = 0.78, *p* = 0.46], or their interaction [*F*_(2, 48)_ = 0.70, *p* = 0.50], which indicates that the training load was well-balanced between the participant groups and training sessions.

**Figure 3:**
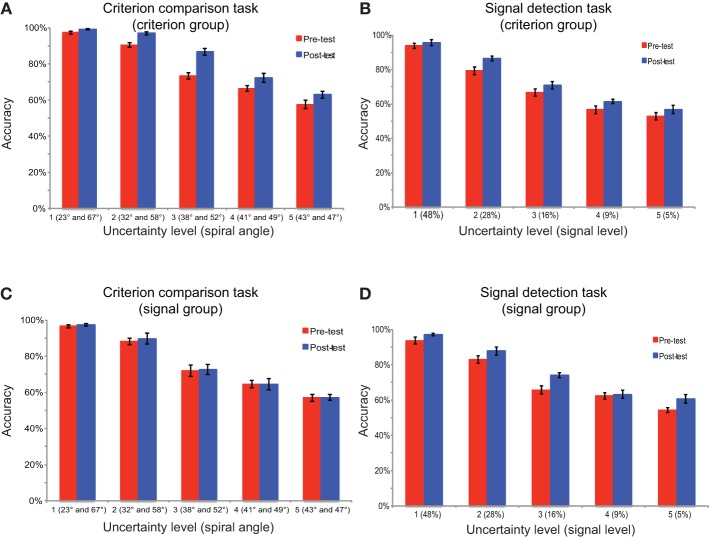
**The categorization accuracy in the test sessions**. The performance is shown for **(A)** the criterion comparison group in the criterion comparison task and the signal detection task, **(B)** the signal detection group in the criterion comparison task and the signal detection task. Error bars represent the standard errors of the means.

To examine participants' training effects, the categorization accuracy was examined for both groups of participants in both tasks and test sessions (Figure 3). A mixed-design, Three-Way ANOVA [test session (pre-test, post-test) × task (criterion comparison task, signal detection task) × participant group (criterion comparison training, signal detection training)] on accuracy revealed a marginal significant three-way interaction effect [*F*_(1, 24)_ = 3.83, *p* = 0.06]; a marginal significant interaction between test session and group [*F*_(1, 24)_ = 3.76, *p* = 0.06]; and a significant interaction effect between task and group [*F*_(1, 24)_ = 15.94, *p* = 0.001]. These results suggested that the two groups of participants have different patterns of learning effects in the two tasks. *Post-hoc* comparisons with Bonferroni correction for simple main effects showed that a significant effect of test session was observed in both tasks for the criterion comparison group (*ps* < 0.01), and in the signal detection task (*p* < 0.01), but not in criterion comparison task (*p* = 0.72) for signal detection group. In summary, there was an asymmetrical transfer of learning between the two tasks. The criterion group improved their accuracies in both tasks, but the signal group only improved their accuracies in their trained task (the signal detection task). Uncertainty level was not included in the overall ANOVA for two reasons. First, we believed that analyzing averaged accuracy across uncertainty levels was informative enough to reveal the learning effects and its transfer across tasks. Second, the uncertainty levels were specifically chosen for each task in a way that they matched in overall difficulty between tasks according to our pilot experiments. The scales of spiral angle and signal level could not be directly compared with each other.

**Figure 4:**
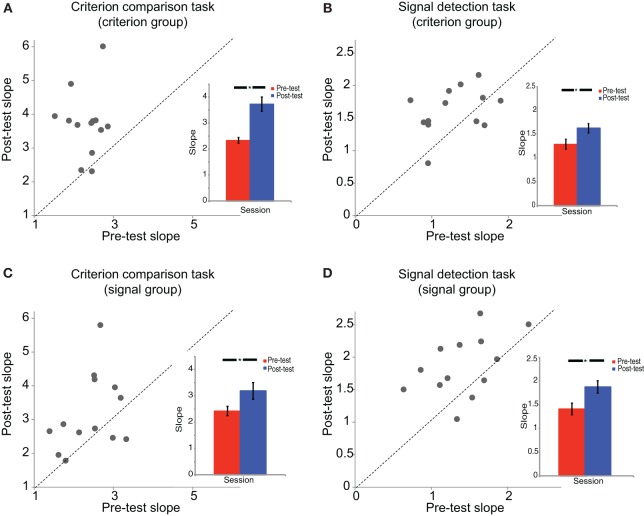
**The slopes of psychometric functions**. The slopes of the psychometric functions are shown for **(A)** the criterion comparison group in the criterion comparison task, **(B)** the criterion comparison group in the signal detection task, **(C)** the signal detection group in the criterion comparison task, and **(D)** the signal detection group in the signal detection task. Each dot in the scatter plot represents one participant's slope of psychometric function in the post-test session vs. the pre-test session. The dashed line is the equal slope line on which the post-test slope is equal to the pre-test slope. The bar figures on the bottom-right are the averaged group results in both the pre-test and post-test. Error bars represent standard errors of the means. ^*^p < 0.05.

The psychometric functions were fitted by the Psignifit toolbox (Wichmann and Hill, 2001) with Cumulative Gaussian functions for the pre-test and post-test sessions. The threshold and slope of each psychometric function were obtained where 50% of the responses categorized stimuli into the concentric group. Due to the different units of the *x* axes of psychometric functions between criterion comparison task (*x* axis stands for spiral angle) and signal detection task (*x* axis stands for signal strength), it was appropriate to conduct two Two-Way ANOVAs separately for the two tasks. We conducted mix-design Two-Way ANOVAs [test session (pre-test, post-test) × participant group (criterion comparison training, signal detection training)] on both the slope and threshold for the criterion comparison task and the signal detection task. For the ANOVA on slope (Figure 4), for the criterion comparison task, there was a significant main effect of test session [*F*_(1, 24)_ = 27.35, *p* < 0.001], but neither significant main effect of participant group [*F*_(1, 24)_ = 0.79, *p* = 0.38] nor their interaction effect [*F*_(1, 24)_ = 2.31, *p* = 0.14]. Similar results were found for the signal detection task [test session: *F*_(1, 24)_ = 21.87, *p* < 0.001; participant group: *F*_(1, 24)_ = 1.84, *p* = 0.19; interaction: *F*_(1, 24)_ = 0.54, *p* = 0.47]. *Post-hoc* comparison with Bonferroni correction showed that both participant groups improved their slopes in both tasks (criterion comparison group: criterion comparison task, *p* < 0.001, signal detection task, *p* = 0.01; signal detection group: criterion comparison task, *p* = 0.015, signal detection task, *p* = 0.001). For the ANOVA on threshold, there were no main effects of test session, participant group, or their interaction in both tasks (criterion comparison task, *ps* > 0.15; signal detection task, *ps* > 0.5).

There was seemingly a contradiction between the categorization accuracy and the slope of the psychometric fitting. However, the measurement of accuracy was related to both the slope and threshold of the psychometric curves. The failure of seeing the transfer effect on the accuracy of the criterion task for the signal group can be attributed to the null effect of the threshold. To specifically examine the training effect on uncertainty reduction, we applied a computational approach to quantitatively model the two types of uncertainty.

### Computational modeling results

There were two main purposes for the computational modeling: to justify the existence of the two types of uncertainty and to examine how the training of each task affected each type of uncertainty. We fitted the behavioral data in the pre-test and post-test sessions separately with all candidate models. If our hypothesis of the existence of the two types of uncertainty was false, the single models were expected to win the comparison; otherwise, the double models were expected to win. The AIC and BIC values of each model in each test session are shown in Table 1. The Double-Simple Model yielded the minimum AIC and BIC values in all test sessions, indicating that this model best characterized the behavioral performance among the four candidate models. Therefore, our results justified the existence of the two types of uncertainty and rejected the alternative hypothesis that only one type of uncertainty existed. Moreover, the model fitting was stable. We correlated the fitted data with the observed data and showed that for criterion comparison group, *r*^2^ = 0.91 for both pre-test and post-test; for signal detection group, *r*^2^ = 0.88 for pre-test and *r*^2^ = 0.91 for post-test. The following analyses were focused on fitting the results of the Double-Simple Model.

**Table 1:** **Model comparison**.

	**AIC**		**BIC**	
	**Pre-test**	**Post-test**	**Pre-test**	**Post-test**
Double-Full	483.96 (12.79)	517.21 (12.81)	431.02 (13.93)	464.38 (13.93)
Double-Simple	462.47 (10.58)	483.24 (10.59)	401.62 (10.59)	422.47 (10.49)
Single-Full	499.60 (11.50)	528.68 (11.50)	465.02 (15.69)	494.22 (15.69)
Single-Simple	468.29 (10.28)	484.91 (10.29)	406.82 (9.70)	423.51 (9.69)

The means and standard errors are shown for the AIC and BIC values from the model fitting.

**Figure 5:**
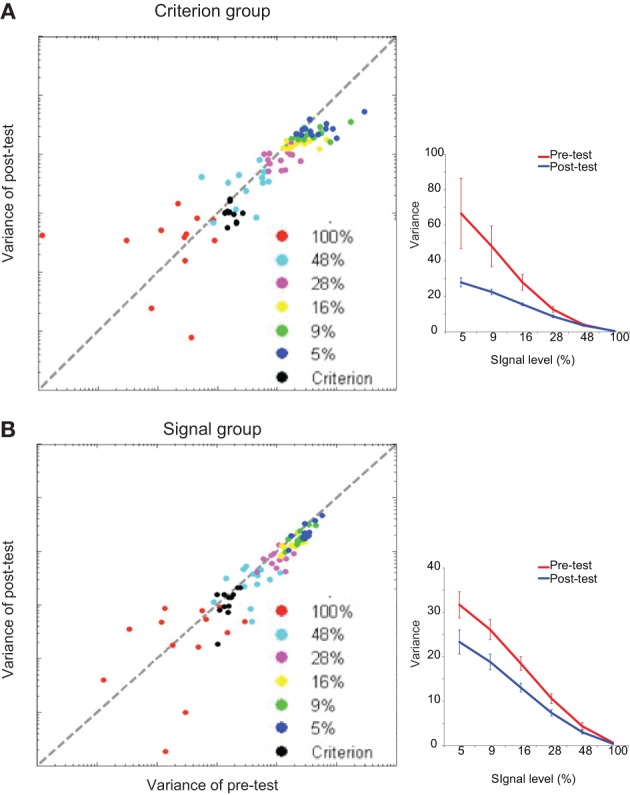
**Model fitting results for the signal uncertainty**. The signal uncertainty is indexed by the variance of perception. The fitting results are shown for **(A)** the individual data and group average of the criterion comparison group, **(B)** the individual data and group average of the signal detection group. Scatter plots show individual results of the model fitting. Each dot denotes one participant's perception variance at one signal strength condition. Error bars represent the standard errors of the means.

**Figure 6:**
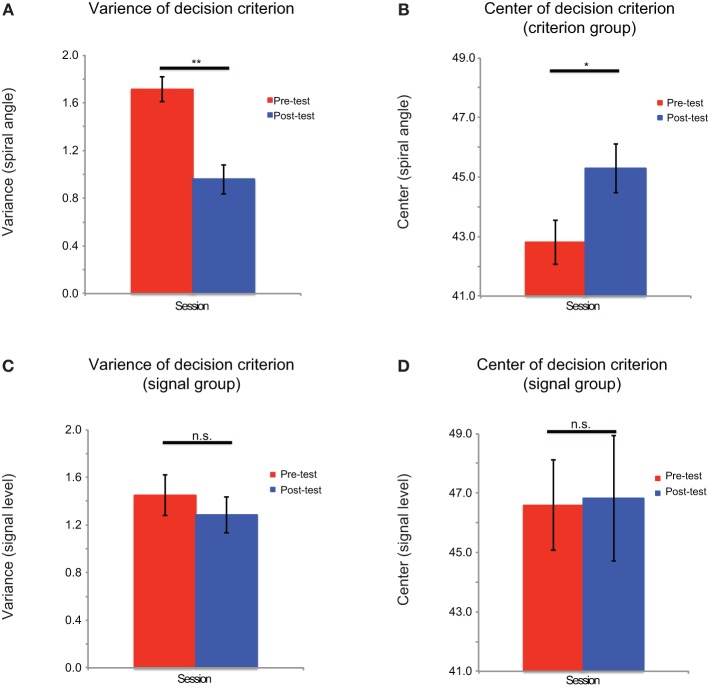
**Model fitting results for the criterion uncertainty**. The criterion uncertainty is indexed by the variance of decision criterion. The fitting results are shown for **(A)** the variance of the decision criterion distribution of criterion comparison group, **(B)** the mean of the decision criterion of the criterion comparison group, **(C)** the variance of the decision criterion distribution of signal detection group, and **(D)** the mean of the decision criterion of the signal detection group. Error bars represent the standard errors of the means. ^**^p < 0.01; ^*^p < 0.05; n.s.: not significant.

For each training group, the variances of g(*p*_*i*_) for the six signal levels in the pre-test and post-test sessions were entered into a repeated measured ANOVA (signal level × test session). There were main effects of the signal level [criterion comparison group: *F*_(5, 60)_ = 16.78, *p* < 0.001; signal detection group: *F*_(5, 60)_ = 83.38, *p* < 0.001] and test session [criterion comparison group: *F*_(1, 12)_ = 5.97, *p* < 0.05; signal detection group: *F*_(1, 12)_ = 26.42, *p* < 0.001]. There was also a significant interaction effect [criterion comparison group: *F*_(5, 60)_ = 4.57, *p* = 0.001; signal detection group: *F*_(5, 60)_ = 15.78, *p* < 0.001]. These results demonstrate that training on both tasks could help the participants to reduce the signal uncertainty of perceptual decisions (Figure 5). The variances of g(*c*_*i*_) in the pre-test and post-test sessions were entered into pairwise *t*-tests. Only the criterion comparison group showed significant changes in the mean and variance of the decision criterion: the mean was shifted toward 45° [*t*_(12)_ = 2.50, *p* < 0.05], and the variance decreased after training [*t*_(12)_ = −4.54, *p* = 0.001] (Figure 6). The results suggest that although signal uncertainty could be reduced by both training tasks, the criterion uncertainty could only be reduced by training on the criterion comparison task. Combined with the behavioral results, we suggest that the reduction in uncertainty of perception played a key role in both tasks, but a reduction in criterion uncertainty was critical to the success of the criterion comparison training. Although the slope of the psychometric curves captured the former uncertainty reduction, it failed to reveal the latter. However, the categorization accuracy was a sensitive index to the reductions of both types of uncertainty. Taken together, our findings suggest that learning-induced uncertainty reduction is task-dependent.

## Discussion

In this study, we investigated how perceptual training modulated task-dependent perceptual uncertainty. The behavioral results showed an asymmetric transfer of learning on categorization accuracy from the criterion comparison task to the signal detection task, but not vice versa. Further analysis with computational modeling revealed that training on both the criterion comparison and signal detection tasks reduced the signal uncertainty, but only the criterion comparison training exhibited a reduction in the criterion uncertainty. These results suggested a possible interpretation underlying the asymmetric learning transfer in the behavioral data. That is, training of fine discrimination on clear global patterns could improve behavioral performance by reducing both the criterion and signal uncertainties. However, training tasks consisting of detecting a global pattern from a noisy background can only reduce the signal uncertainty that was indexed by perceptual variance. Our findings also extended our understanding of the mechanisms for the uncertainty representation in perceptual decision-making and suggested necessity of clear classification of uncertainty type.

Our previous study identified brain areas in which the fMRI activity was correlated with uncertainty levels in the criterion comparison and signal detection tasks (Li and Yang, 2012). While the areas responsible for performance monitoring represented the decision uncertainty in both tasks, the dissociable cortical networks were also identified. Uncertainty modulated activity in the area related to rule retrieval in the criterion comparison task and the higher visual processing areas in the signal detection task. Taken together with the present results, these findings suggest that training on both the criterion comparison and signal detection tasks could improve the efficiency of high level visual processing, which therefore would provide less ambiguous sensory information to the decision-related brain networks. However, only the training on the criterion comparison task, where fine discrimination is required, could improve the rule-retrieval process in the categorization task, which lead to a reduction in the uncertainty for the decision criterion. Future investigations that combine the perceptual training paradigm and neuroimaging technique are required to further elucidate the neural mechanism(s) for this interaction between learning and uncertainty modulation.

The modeling results in uncertainty reduction were in agreement with the behavioral improvements observed before and after the training. The behavioral results on overall accuracy that showed improved performance on the signal detection task for both participant groups were accompanied with a reduction in criterion uncertainty based on the fitting of their behavioral data. On the other hand, the performance of the criterion comparison task was improved only for the participants who were trained on it, and this effect was accompanied with reduced criterion uncertainty after training. These findings suggested that a reduction of criterion uncertainty played a key role in the fine discrimination task, but the improved sensitivity to the sensory evidence is not sufficient. Moreover, in terms of training efficiency, the asymmetric transfer of learning and the subsequent task-dependent uncertainty reduction should be considered in the designs of new perceptual training paradigms for clinical purposes.

A similar asymmetric transfer of learning in a Gabor orientation discrimination task has been found in a previous perceptual learning study by Dosher and Lu (2005) (Just to increase the connect between two graphs). Their results showed that training on clear stimuli could improve the filtering of external noise and enhance the perceptual template. Therefore, the training effect can be transferred to the task with noisy stimuli. However, the training effect on the noisy stimuli could not be generalized to the clear ones because the signal and external noise would be amplified together due to the training (Dosher and Lu, 1998). Only training on the clear stimulus could effectively reduce the internal noise of the neural system. Our results are in agreement with their findings and further generalized their results to the high level visual perception. We showed that practicing on noiseless global patterns could improve the ability of filtering out external low level noise (i.e., random local dipole orientation). Furthermore, our results also provided evidence that a training-induced reduction in criterion related decision uncertainty could contribute to the observed learning effect on clear patterns. The improvement was evident by the reduced variance in decision criterion for the criterion group but not the signal group. In relation to Dosher and Lu's modeling work, our findings provide an alternative explanation about the mechanism of internal noise reduction. That is, in addition to the improved perceptual processing, the decision-related factors can be modified by perceptual training, which leads to improved performance for perceptual decisions (Law and Gold, 2008; Xiao et al., 2008).

Finally, our results were unlikely due to differences in task difficulty between the criterion comparison and signal detection tasks. We adaptively adjusted the stimuli and matched the performance across training sessions and participants. Furthermore, we adopted a single task framework to investigate both the criterion and signal uncertainties, ruling out the possible confounding factors such as task designs and qualitative differences in the stimuli. In summary, our findings provide evidence that the uncertainty in perceptual decision-making processes can be reduced with training but that the transfer of the uncertainty reduction exists only from the criterion to signal uncertainty.

### Conflict of interest statement

The authors declare that the research was conducted in the absence of any commercial or financial relationships that could be construed as a potential conflict of interest.

## Footnotes

^1^In our pilot fitting process with Full Model, the variance in each condition showed an exponentially decreasing pattern (or a linearly decreasing pattern given a logarithmic-y axis). Additionally, it was unlikely that variances for different signal strength conditions were all independent to each other. Therefore, based on linear logarithmic pattern of variances in the pilot data, we fitted simple model by using an exponential function denoting the variances for different signal strength conditions.
